# Treatment of Furcal Perforation of Primary Molars with ProRoot MTA versus Root MTA: A Laboratory Study

**Published:** 2013-05-01

**Authors:** Roza Haghgoo, Farid Abbasi

**Affiliations:** 1 Department of Pediatric Dentistry, Dental School, Shahed University of Medical Sciences, Tehran, Iran; 2 Department of Oral Medicine, Dental School, Shahed University of Medical Sciences, Tehran, Iran

**Keywords:** Dental Leakage, Endodontics, Mineral Trioxide Aggregate, Sealing Material

## Abstract

**Introduction:**

Furcal perforations are one of the most challenging causes of endodontic failures. Several materials including MTA have been used for non surgical repair of these perforations. The aim of this study was to compare treatment outcome of furcal perforation treatment in primary molars using Root MTA and ProRoot MTA.

**Materials and Methods:**

This *in vitro* study was conducted on 54 primary first molars that were randomly divided into the two experimental groups of 24 teeth each and two control groups (*n*=6). After preparation of access cavities, perforations were made and the perforation areas were repaired using either Root or ProRoot MTAs. After staining and preparation of mesiodistal longitudinal sections, dye leakage was measured using a stereomicroscope. The data was analyzed by the Mann Whitney statistical test. Significant level was set at 0.05.

**Results:**

The data indicated that the dye leakage of ProRoot MTA is significantly lesser than Root MTA (P=0.001).

**Conclusion:**

ProRoot MTA showed good sealing ability in repairing furcal perforations of primary molars.

## 1. Introduction

One of the most important causes of failure in endodontic treatment is tooth perforations at different sites among which furcal perforations have the worst prognosis. Perforations of the pulp chamber space lead to inflammatory response in periodontium that may cause irreversible destruction of the periodontal ligament or even tooth loss (1). Early treatment of perforations is therefore necessary for tooth retention (2). Several materials have been suggested for repairing of furcal perforations such as amalgam, gutta-percha, cavit, composite resin, MTA, glass ionomer, super EBA, calcium hydroxide, and calcium enriched mixture (3). The ideal material for treatment of root perforations must be non-toxic, radiopaque, bacteriostatic/bactericidal and unresorbable (4-5).

ProRoot MTA has an alkaline pH (5), and studies have shown that its performance in microleakage protection is superior to amalgam, IRM, and super EBA (3, 6, 7). MTA has a low cytotoxicity (8), and good antibacterial properties (9, 10). It is biocompatible and can induce osteogenesis and odontogenesis (7, 11-13).

Recently, Root MTA has been introduced (Tabriz, Iran) and shown similar characteristics to ProRoot MTA in-vitro and in-vivo (14-16). Repairing of furcal perforations using Root MTA has previously been evaluated (1-3, 17-19). However, none of these two materials have been used in primary molars. Accordingly, the purpose of this *in vitro* study was to compare the sealing ability of Root MTA and ProRoot MTA in repairing furcal perforations in primary molars.

## 2. Material and Methods

In this in vitro study, fifty-four maxillary and mandibular primary molars with completely formed roots were used. The furcation areas were healthy and had normal anatomy. The teeth were randomly divided into two experimental groups of 24 teeth each (12 upper molars and 12 lower molars, in each group), a negative control group (without perforations, *n*=3) and a positive control group (perforations without repair, *n*=3). The teeth were disinfected in 5% sodium hypochlorite solution for 30 min, then rinsed with water and preserved in saline. Roots of molars were amputated in the middle third area using a tapered diamond stone. An endodontic access cavity was made in each tooth using a round diamond bur (D&Z, Wiesbaden, Germany) in a high-speed handpiece. Floor of the pulp chamber, canal orifices and apices of amputated roots were then etched with 37% phosphoric acid and following application of bonding agent, canal orifices and apices of amputated roots were sealed using composite resin.

Perforations measuring 1mm in diameter were then created in the furcal areas using a round diamond bur (D&Z Wiesbaden, Germany). The teeth were rinsed with water and dried with an air spray. The width of perforation in all teeth was the same, but depth of perforations was different depending on the existent morphological thickness of dentin and cementum in the floor of pulp chamber. At this stage, depths of perforations were checked using a periodontal probe and if this was not within 1-1.5 mm range, the tooth was excluded. Root MTA (Root MTA, Manufactured under IR Patent: 38720) and ProRoot MTA (ProRoot MTA, Dentsply Tulsa, Tulsa, OK, USA) were prepared according to manufacturer’s instructions, placed into the perforation and gently condensed with a paper point.

Throughout the experiment, a moist cotton pellet was placed between the roots in an attempt to hydrate the tooth and repair materials. A moist cotton pellet was also placed on MTAs and the access cavities were sealed with a dressing. The samples were then placed in an incubator for 72 hours in 37^º ^C and 95% humidity. All of the teeth were then covered with 2 layers of nail varnish covering an area except for 2 mm around the perforation.

The teeth were placed in laboratory tubes (3 teeth in each tube) and immersed in 8 mL of fuchsin solution. The tubes were then centrifuged 300 times per minute for 15 minutes. The samples were removed from the tubes, placed passively into fuchsin for 24 hours, and were then rinsed with water. The samples were then placed in clear acrylic blocks and were sectioned mesiodistally parallel to the long axis of the teeth. Using a stereomicroscope (Carton, SCW-E, Thailand) dye leakage was measured. Measurements were carried out blindly by three independent observers. As the height of perforations of the teeth was different, dye penetration was calculated based on the length of perforation in each tooth. The cases which showed difference between the results obtained by the observers were re-evaluated.

## 3. Results

Of the three maxillary and mandibular molars in the positive control group, complete dye penetration was observed in all of the samples. Samples in the negative control group showed no dye penetration.

**Figure 1.**
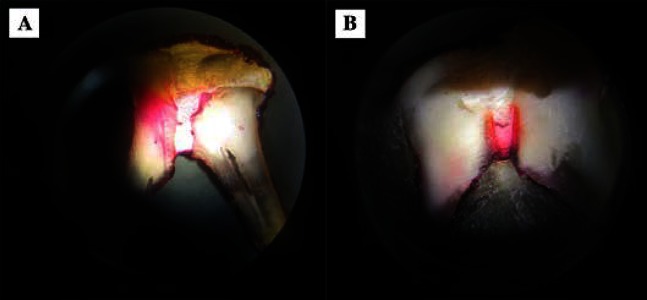
A) Repair of perforation with ProRoot MTA; B) Repair of perforation with Root MTA

There were no significant differences between maxillary molars and mandibular molars repaired with either ProRoot MTA or Root MTA (Table 1). However, there was a significant difference (P=0.001) between Root MTA and ProRoot MTA in repair of furcal perforations (Mann Whitney *U* test) (Table 2), (Figures 1A, 1B).

**Table 1.** Comparing maxillary and mandibular molars repaired with ProRoot and Root MTA

MTA	Jaw	N	Mean Rank	Sum of Ranks
**ProRoot**	Maxilla	12	10.46	125.50
**ProRoot**	Mandible	12	14.54	174.50
**Root**	Maxilla	12	11.71	140.50
**Root**	Mandible	12	13.29	159.50

**Table 2.** Comparing dye leakage with ProRoot and Root MTA

MTA (n=24)	Mean Rank	Sum of Ranks
**ProRoot**	14.42	346.00
**Root**	34.58	830.00

## 4. Discussion

A primary molar tooth with inadequately repaired furcal perforation has a poor prognosis. Such perforations can be adequately treated surgically and non-surgically. The ideal material for repairing furcal perforations must be non-toxic, radiopaque, bacteriostatic/bactericidal (1). MTA has been suggested for repair of perforations, since the sealing ability of MTA is superior to amalgam and super EBA (2). As there are different brands of MTA, we tested Root MTA with an original brand for their sealing ability as perforation repair materials; Root MTA showed significant dye leakage. In a previous study by Labbaf et al., both Root MTA and ProRoot MTA were condensed by one operator under similar standardized conditions (18). It was shown that Root MTA was not condensed evenly, and the ratio of powder to liquid, temperature and air entrapped into the mass, can affect the form of the material. Therefore, inadequate condensation of Root MTA may be related to particle size, and it is possible that remixing the material can lead to a more even mixture that can adequately seal the perforation area.

In a study by Bidar et al. furcal perforation treatment with Root MTA, ProRoot MTA and one coat bond was compared (2). The results indicated that there were no significant differences between sealing ability of Root MTA, ProRoot MTA and one coat bond. However, since the study was carried out on permanent molars and stained using Indian ink, this may explain why the results differ compared to ours. In another study (19), histological assessment of furcal perforation repair using Root MTA and ProRoot MTA in dog's mature teeth was performed. The results indicated that Root MTA was a suitable agent for sealing of furcal perforations. However, differences in histology of dog and human teeth may affect the results of this study.

## 5. Conclusion

Based on the results of this *in vitro* study Root MTA is not a suitable substitute for ProRoot MTA in repairing of furcal perforations of primary molars.
